# Meeting Review: Joint Prince Henry's Institute of Medical Research and Monash Institute of Reproduction and Development Symposium – Reproductive Genomics

**DOI:** 10.1002/cfg.151

**Published:** 2002-04

**Authors:** Gerard Gibbs, Moira K. O'Bryan

**Affiliations:** 1 Monash Institute of Reproduction and Development Monash University Melbourne Australia; 2 Monash Institute of Reproduction and Development Monash University 246 Clayton Road Clayton 3168 Australia


**Comparative and Functional Genomics**
*Comp Funct Genom* 2002; **3**: 205–208.
Published online 12 March 2002 in Wiley InterScience (www.interscience.wiley.com). **DOI:** 10.1002/cfg.151

**Feature**

### Meeting Review: Joint Prince Henry's Institute of Medical Research and Monash Institute of Reproduction and Development Symposium – Reproductive Genomics

### Monash Medical Centre, Melbourne, Australia. December 5–6, 2001

Gerard Gibbs and Moira K. O'Bryan*

*Monash Institute of Reproduction and Development, Monash University, Melbourne, Australia.*

**Correspondence to:*
*Monash Institute of Reproduction and Development, Monash University, 246 Clayton Road, Clayton 3168, Australia.*
*E-mail: moira.obryan@med.monash.edu.au*

Monash Institute of Reproduction and Development (MIRD) and Prince Henry's Institute of Medical Research (PHIMR) are institutes with a traditional focus on reproductive biology. Located in Melbourne, they have expertise ranging from male and female fertility/infertility, hormonal regulation, pre-implantation biology and screening, sudden infant death syndrome, cancers of the reproductive tract, early human development, assisted reproductive technologies, endangered species conservation, and more recently, the development and use of embryonic stem cells for strictly non-reproductive purposes. This environment formed the perfect setting for an international symposium on reproductive genomics – a relatively new discipline, which, judging from the high caliber of data presented and the number of attendees, is being embraced emphatically.

The meeting was opened by **the Honourable John Brumby (The Victorian State Government Treasurer and Minister for State and Regional Development)** who signaled a bright future for Australian (and Victorian in particular) biotechnology and its underpinning basic research, through a strong Government financial and legislative commitment to place Victoria within the top five biotechnologies regions world wide by 2010. The success of such a venture however, is solely dependent on attracting and retaining the 'best and brightest' Australian researchers through improved salary conditions and support - a point that has long been heralded by organizations such as The Australian Society for Medical Research, but difficult to meaningfully implement in the face of biotechnology giants based in the regions such as those around San Diego, San Francisco Bay and Boston. This commitment and the possibility of combining the very attractive Australian lifestyle with improved work conditions was, however, enthusiastically received.

The scientific sessions were started by **Austin Cooney (Baylor College of Medicine, TX)** who vividly highlighted the value of the mouse as a model organism and the necessity for meticulous phenotypic analyses in order to grasp the underlying biology. This theme was echoed by **Margaret Jones (PHIMR, Australia)** and **David Bowtell (Peter MacCallum Cancer Institute, Australia)** later in the day. Austin presented his lab's work on germ cell nuclear factor (GCNF) and the various *cre*-*lox* mouse models they have constructed to unravel its importance in male and female reproductive biology. Male and female germ line specific *gcnf* deletions were produced driven by *sycp1* and *zp3* promoter linked *cre* respectively. He showed that despite the implementation of impressive molecular biology and the success of ROSA assays, inactivation of the *gcnf* locus driven by *sycp1* promoter linked *cre* occurred in only 10% of male germ cells. The *zp3* driven excision was, however, very successful, yet ovarian histology appeared normal and females were fertile. Fortunately, through the thorough (and at times no doubt heart breaking) efforts of Austin's post-docs, it was noticed that the estrous cycle (and specifically diestrous) of

Copyright © 2002 John Wiley & Sons, Ltd.

knockout mice was approximately twice that of control mice confirming that, as suspected, *gcnf* is indeed required for 'normal' ovarian function.

**Margaret Jones** presented a review of many years of analysis on the aromatase knockout mouse (*arko*) established by Evan Simpson's laboratory and the roles of aromatase, both obvious and subtle, in female and male fertility. Further, Margaret presented new data on the success of *arko* mice treated by intracytoplasmic sperm injection.

**David Bowtell** presented his laboratory's work on the *sina/siah* family of molecules in development with an emphasis on the mechanism of meiotic metaphase arrest and subsequent male infertility seen in the *siah1a* knockout mouse.

**Marilyn Monk's (Institute of Child Health, UK, and MIRD, Australia)** research and presentation revolved around the hypothesis that embryonic genes are re-expressed in cancer cells i.e., both cell types undergo genome wide demethylation, are immortal, undifferentiated and invasive. Such proteins/genes would represent ideal targets for the treatment of cancer by DNA vaccines. In order to explore this hypothesis the Monk group effectively had to start from 'scratch' as existing expression libraries and microarrays are derived from somatic cells and, by definition, do not contain embryo-specific sequences – and most certainly not of human origin. In order to overcome obvious limitations in obtaining tissue, cDNA libraries were derived from single human oocytes and single embryos from all of 2-cell, 4-cell and 8-cell stages, blastocytes and primordial germ cells (PGCs) and amplified. Embryo-specific sequences were obtained using differential display by comparison to a panel of 10-week fetal somatic tissues. Marilyn described the preparation of these unique libraries and the isolation of 3 previously unknown embryonic sequences that are re-awakened in human tumors. This investigation also highlighted the value of research dollars spent on non-lethal conditions, such as infertility, for perhaps more physically taxing diseases such as cancer.

**Gary Hime (University of Melbourne, Australia)** presented his work, and a literature review, on *Drosophila* spermatogenesis and its value as a model for mammalian systems. Specifically, there is a high conservation of disease causing genes between flies and humans ($\sim$62%), they have short life cycles, are easily genetically manipulated and are amenable (from both a labour and dollar perspective) to large, genome-wide analyses. Models outlined included four-wheel drive (*fwd*), escargot and several novel sequences obtained by the Hime laboratory while looking for testicular stem cell specific sequences.

**Mary Stevens (Bayer Biotechnology, Berkeley, CA)** gave an introductory presentation on Bayer's research into viral delivery systems for their use in the production of transgenic animals. Specifically she spoke about their experiences with the adenovirus and adeno-associated virus (AAV) systems and their use in models of asthma and cancer. While not focusing on any reproductive disorders, the asthma and cancer systems were seen as forerunners for future research. By comparison to the adenovirus system (ADEasy System), the AAV system was found to produce longer term (perhaps permanent), but lower level, more physiological over-expression, but was limited by a relatively small insert size of $\sim$4.5 kb, difficult construction and unregulated production of largely soluble proteins. Both systems appeared to have advantages over more traditional methods of transgenic mouse production for addressing specific biological questions.

The potential value of viral delivery systems is beginning to be realized in the clinic, as outlined in the presentation by **Leland Chung (Emory University, GA)**. Leland presented his group's results on the use of adenovirus-based vectors in combination with more traditional therapies such as anti-angiogenic drugs, antibody and growth factor conjugated toxins and radiation therapy for the treatment of prostate cancer. These investigations demonstrated that targeted gene therapy was able to limit metastasis, however Leland also cautioned that to be more effective, improvements in viral targeting need to be made.

The application of microarrays to the study of reproductive disorders has been whole-heartedly and productively embraced by reproductive biologists. These techniques provide a detailed picture of complex genetic diseases and have emerged as an important research tool, which is being extended into clinical diagnosis. **Steve Smith (Cambridge University, UK)** presented his work on human female infertility and outlined the significant medical problems, and the lack of data, surrounding the time of pre-implantation. The lack of data is caused by the difficulties in obtaining human tissues and the increased complexity of human implantation compared to mice, and as such, the paucity of suitable model systems. Steve's lab has used the

Copyright © 2002 John Wiley & Sons, Ltd. 

Affymetrix array system (representing ~90% of the human genome) and subsequently tailored membrane-based arrays to assess differences between individual patient samples of proliferative and secretory endometrium in order to understand the origin of various types of menstrual/angiogenesis based conditions. Using this method, 11 marker genes were identified. He noted that the variation between patients was often greater than the differences between disease states when looking at an individual gene level. As such, it is imperative for researchers to look at cassettes of gene expression, including the level of expression, in order to get a grasp on the underlying biology. A systems biology approach to the understanding of disease through the use of microarray expression profiles may require complex analyses and the recruitment of traditionally mathematical minds.

**Nabeel Affara (Cambridge University, UK)** has very successfully used glass slide based arrays of largely testis-specific sequences. They have used four gene sets: 1. Testis-specific sequence libraries from all of human, mice and pig, produced by subtraction of testis RNA with RNA from somatic tissues (1300, 5400, and 4700 clones respectively); 2. Mouse testis RNA subtracted with $Sxr^b$ mouse testis RNA (which lack germ cells) (2000 clones); 3. the McCarrey/Eddy gene set of germ cell transcripts (~8000 clones) and 4. the National Institute of Ageing (NIA) mouse embryo gene set (~15 000 clones or ~50% of the mouse transcriptome). These arrays have been used to assess expression differences between various RNA samples including those derived from human somatic tissues, testicular cell lines (GC1, GC2, TM3 and TM4) and mouse testes of defined ages to identify truly testis-specific sequences, and those specifically involved in the first wave of spermatogenesis. Reference probes for all were derived from either adult mouse or human testis RNA. Importantly, all analyses were done using both combinations of fluorochromes i.e., test and reference cDNAs labeled with both fluorochromes. Only those showing a significant fold increase/decrease with both labels were taken as meaningful.

**Peter Koopman (Institute of Molecular Bioscience, Australia)** presented his laboratory's work on the use of microarrays for the discovery of genes (and transcriptional differences) involved in either testis or ovary development. Membrane arrayed samples consisted of male versus female (and *vice versa*) subtracted and normalized libraries generated using suppression PCR; the NMUR library (Greenfield/Soares) and unselected cDNA sets derived from NIA and RIKEN sequences. Genes involved in female or male sex determination were identified from critical stages of embryogenesis (i.e., 11 days or 13 days), by analysis of differentially expressed RNA on DNA microarrays. Sequences were analyzed using various bioinformatics programs and results validated, and the expressing cells determined, using whole-mount *in situ* hybridization. Results were presented for vanin-1 and several novel sequences. Vanin-1 was shown to be localized at the surface of Sertoli cells and be involved in testis development and cord formation through the use of the urogenital ridge co-culture assay.

**Vince Harley (PHIMR, Melbourne)** addressed the accumulating knowledge of genes critical in the determination of 'maleness', a theme that was rather amusingly expanded upon from an evolutionary perspective by **Jenny Graves (The Australian National University, Australia)**. Through genomic approaches, such as those presented by **Peter Koopman**, and more traditional molecular biological/transgenic approaches, the field is moving rapidly towards an understanding of the critical events in sex determination. The Y chromosome may not be the sex determining 'powerhouse' it was once purported to be, but rather an 'whimpish' remnant of chromosomal history slated for destruction.

As a further dimension to the field of sex determination, **Eric Vilain (University of California Los Angeles, CA)** presented his work on dosage-sensitive sex reversal, and specifically *WNT-4*. Further, he presented some of the consequences of the misguided, although well meaning, intentions of surgeons operating on inter-sex human patients, a point that vividly highlighted the necessity for increased communication between medical researchers, medical staff and patients.

The proteomics session began with **Jenny Harry (Proteome Systems, Australia)**, who outlined their strategy for high throughput 2D gel analysis and presented some of their results on body fluid and tissue derived biomarkers of ovarian cancer. Treatment of ovarian cancer is critically dependent upon early diagnosis (>90% survival rate), but is rarely diagnosed until it has reached late stage (>85% of cases). Jenny also outlined the development of their ProteoChip (membrane immobilized spots) which will allow for sample storage and subsequent re-analysis.

Copyright © 2002 John Wiley & Sons, Ltd.

**Paige Hilditch-Maguire (Ciphergen, Australia)** presented their work on a surface enhanced laser desorption and ionization (SELDI) platform and the success of this machine in searching for disease progression markers including those for ovarian carcinomas and prostate cancer.

**Peter Hudson (CSIRO, Australia)** provided an overview of the emerging field of protein arrays and their potential applications in the discovery of biomarkers, and in the development of protein chips as point of care diagnostic devices. Techniques for the development of stable protein chips are still being developed, however, by coupling antigens or dimers and trimers of recombinant antibody fragments to hydrated surfaces, the promise of protein chips as a complement to DNA microarrays is becoming a reality.

**Chris Goodnow (The Australian National University, Australia)** gave an elegant illustration of the strategy for, and value of, large-scale ENU mutagenesis programs by effectively dissecting the T cell differentiation pathway using mice with names such as 'Buffy', 'Willow' and 'Xander'. A theme that was expanded upon by **Chris Ormandy (Garvan Institute, Australia)** who has used ENU mutagenized mice from Chris Goodnow's colony to identify genes involved in mammary gland development and carcinogenesis. After an initial screen using mutagenized C57BL/6 mice, the Ormandy lab has subsequently moved onto using sensitized mouse lines such as those carrying the SV40 T antigen or sensitizing transgenes (e.g., *myc* and *wnt*) in order to recover a greater percentage of cancer promoting mutations.

If there was any doubt as to the value of the mouse as a model system, its genetic potential was hammered home by **Simon Foote (The Walter and Eliza Hall Institute, Australia)** who has used mouse strain differences to map genetic modifiers of disease. Specifically, his laboratory has used strain susceptibility differences to *Leishmania major* or tumorigenesis to map quantitative trait loci (QTL). Once identified, candidate regions were validated by the production of BAC transgenic mice to assess disease resistance on an otherwise susceptible background. Importantly, all regions mapped by the laboratory have been QTLs. They have never isolated a Mendelian single gene trait, thus underscoring the value of large linkage based analyses, where possible, over the candidate gene approach.

The value of the translation of high quality animal model research into a human setting was emphasized by the work presented by **Ken McElreavey (Pasteur Institute, France)** on Y chromosome deletions and haplogroups in spermatogenic failure, high sperm counts and twinning. Further, the value of the observant clinical eye and memory was left in no doubt. Most excitingly, Ken presented the discovery (in collaboration with **Prof. Golubovsky, St Petersburg, Russia**) of families with a Y chromosome linked predisposition to twinning (and miscarriage). Until now, the origin of all twinning was believed to be either multiple ovulation or zygote/early embryo cleavage. Amazingly these families were spread across four countries, shared the same surname (and indeed Y chromosome) and could be traced back to a common ancestor in 17$^{th}$ Century Scotland. Currently the mechanism by which this happens is unknown.

Other highlights of the meeting included the presentations by **Paul Hertzog (MIRD, Australia)** on chromosome 21 genes and diseases. His group has successfully constructed several chromosome 21 gene mouse models, which have shed light not only upon Down Syndrome pathology, but also on the etiology of more widespread conditions including viral susceptibility (*ifnar1* and *infar2* knockout mice), neuronal degeneration (*ets2* knockout) and kidney degeneration (*adamts-1* knockout). **Carina Dennis (Nature Publishing Group, Washington DC)** discussed the considerable problems associated with archiving and disseminating the massive amounts of data generated by genomic-based programs and presented some potential solutions. **Andrew Elefanty (Walter and Eliza Hall Institute and MIRD, Australia)** and **Martin Pera (MIRD, Australia)** presented a front-line view of embryonic stem cell research and its ultimate potential for human therapies. A session that was nicely followed up by **Bob Williamson (Murdoch Children's Research Institute, Australia)**, who presented a potential road map through the minefield of ethics associated with stem cell technologies and a look back at the path taken for reproductive technologies.

Finally the meeting was concluded with a presentation by **John Mattick (Institute of Molecular Biosciences, Australia)** who gave his perspectives on the value of the human genome and some potential answers to the lower than expected gene number found for humans. His fascinating ideas and data on non-coding RNAs and their role in all levels of gene, mRNA and protein regulation left the audience with no doubt that genome research has a long and glorious path ahead of it.

Copyright © 2002 John Wiley & Sons, Ltd.

